# Epigenetic regulation of *Plasmodium falciparum* clonally variant gene expression during development in *Anopheles gambiae*

**DOI:** 10.1038/srep40655

**Published:** 2017-01-16

**Authors:** Elena Gómez-Díaz, Rakiswendé S. Yerbanga, Thierry Lefèvre, Anna Cohuet, M. Jordan Rowley, Jean Bosco Ouedraogo, Victor G. Corces

**Affiliations:** 1 Department of Biology, Emory University, 1510 Clifton Road NE, Atlanta, GA 30322, USA; 2 Institut de Recherche en Sciences de la Santé (IRSS), 01 BP 171 Bobo Dioulasso, Burkina Faso; 3 Maladies Infectieuses et Vecteurs: Écologie, Génétique, Évolution et Contrôle (MIVEGEC, UM -CNRS 5290-IRD 224), Centre IRD, 34394-Montpellier, France

## Abstract

*P. falciparum* phenotypic plasticity is linked to the variant expression of clonal multigene families such as the *var* genes. We have examined changes in transcription and histone modifications that occur during sporogonic development of *P. falciparum* in the mosquito host. All *var* genes are silenced or transcribed at low levels in blood stages (gametocyte/ring) of the parasite in the human host. After infection of mosquitoes, a single *var* gene is selected for expression in the oocyst, and transcription of this gene increases dramatically in the sporozoite. The same PF3D7_1255200 *var* gene was activated in 4 different experimental infections. Transcription of this *var* gene during parasite development in the mosquito correlates with the presence of low levels of H3K9me3 at the binding site for the PF3D7_1466400 AP2 transcription factor. This chromatin state in the sporozoite also correlates with the expression of an antisense long non-coding RNA (lncRNA) that has previously been shown to promote *var* gene transcription during the intraerythrocytic cycle *in vitro*. Expression of both the sense protein-coding transcript and the antisense lncRNA increase dramatically in sporozoites. The findings suggest a complex process for the activation of a single particular *var* gene that involves AP2 transcription factors and lncRNAs.

*Plasmodium falciparum* is the etiological agent responsible for the most severe form of human malaria, an infectious disease responsible for at least half a million deaths and 200 million clinical cases each year, and for which there is currently no effective vaccine^1^. This protozoan parasite has a two-host life cycle that involves humans and *Anopheles* mosquitoes. Malaria parasites replicate by asexual multiplication in the mammalian host, in liver hepatocytes and red blood cells, and both sexually and asexually in mosquitoes. The parasite journey in its vector starts when a mosquito ingests *P. falciparum* gametocytes from the blood of an infected human host. Fertilization generates diploid zygotes that initiate meiosis within 1–2 hr. Sixteen to thirty hours post-infection, zygotes become motile ookinetes that cross the midgut epithelium and round up on the basal side of the midgut, forming protected capsules called oocysts. Over the next 10 days, parasites undergo multiple rounds of mitosis to produce thousands of sporozoites that are released in the mosquito body cavity about 2 weeks post-infection and migrate to the salivary glands. Parasite development in the vector is completed when the sporozoites are injected with the mosquito saliva into the next human host.

*P. falciparum* has developed an extensive degree of adaptive phenotypic plasticity optimizing transmission between the human and mosquito hosts. In humans, parasite life cycle progression occurs through coordinated waves of gene expression^2^,^3^,^4^, and a similar transcription switch seems to occur in the mosquito host^5^. Experiments carried out with the blood stages of the parasite show a correlation between changes in post-translational modifications of histones (hPTMs) and stage-specific transcription programs^6^,^7^. In *Plasmodium*, like in other organisms, H3K9ac and H3K4me3 are linked to transcription and localize at active promoters, whereas H3K9me3 is a repressive modification that tends to localize in heterochromatic regions, and is associated with gene silencing. Although histone modifications are involved in the regulation of gene expression, their roles appear in most cases, secondary to other molecules and cis-sequences^8^,^9^. The causal involvement of hPTMs in *Plasmodium* life-cycle associated transcriptional transitions has not yet been demonstrated, except for a recent report that shows the essential role of histone deacetylase 2 (PfHda2) in regulating virulence gene expression and gametocyte conversion^10^. Other than hPTMs, recent studies have shown that AP2 transcription factors play key roles in various *Plasmodium* stage-transitions^11^,^12^,^13^. Yet, the contribution of stage-specific transcription factors to gene regulation and cellular memory in *P. falciparum* remains poorly understood, particularly for certain stages of the parasite life-cycle.

Adaptive phenotypic plasticity in *Plasmodium*, and therefore its ability to respond rapidly to current conditions in the host, is tightly linked to the variant gene expression of a number of gene families involved in processes such as antigenic variation, red blood cell invasion, solute transport, and sexual differentiation^14^,^15^. These genes show clonally variant gene (CVG) expression, such that individual parasites having identical genomes and under the same environment can maintain a variant gene in a different transcriptional state and this state can be transmitted to the next generation. The best described are the multicopy *var, rifin, stevor, surfins*, and *Pfmc-2TM* CVG families, which encode antigens expressed at the surface of infected erythrocytes^16^. Among these, *var* genes encode Erythrocyte Membrane Protein 1 (PfEMP1), which is a critical virulence factor for malaria. Each *P. falciparum* parasite has approximately 60 different *var* genes, only one of which is expressed at a time by the clonal parasite population in the infected red blood cells^17^,^18^. The variegated expression of these genes has been shown to correlate with alterations in histone modifications, mainly H3K9me3 and H3K9ac, and these chromatin states can be epigenetically inherited^19^,^20^,^21^. Recent evidence suggests that sense and anti-sense long non-coding RNAs can also regulate *var* gene expression^22^,^23^,^24^.

The passage through the mosquito drastically reduces malaria parasite populations, and can also attenuate parasite virulence during infection of the human host^25^,^26^. Therefore, the parasite mosquito stages represent an important target for interventions aimed at blocking disease transmission. Despite this, the contribution of epigenetic changes and transcription factors to the regulation of phenotypic plasticity and *var* gene expression in *P. falciparum* during its life cycle in the mosquito and the implications for malaria epidemiology remain unknown. Filling this gap in our knowledge is critical. Deciphering the mechanisms of gene regulation across the complete life cycle of the parasite will inform on how successfully *P. falciparum* adapts to the different environments it encounters in each of the two hosts. This information can then be used to identify the most appropriate life cycle stage to be targeted for the development of antimalarial strategies.

Here we examine the transcriptional and epigenetic changes that take place in *P. falciparum* during the life cycle in its natural mosquito host. We experimentally infect *An. gambiae* in the laboratory using blood from malaria-infected human volunteers in Burkina Faso, a malaria endemic transmission area of West Africa. This approach best mimics the malaria infection process occurring in nature, allowing us to interpret the results in an ecologically-relevant context of the disease. We first conducted RNA-seq on four independent blood samples from malaria infected donors containing a mixture of gametocyte and ring (also named early ring-form trophozoites) stage parasites. We also performed RNA-seq on oocyst and sporozoite stages of the parasite life cycle in the mosquito. Next, we carried out ChIP-seq analyses of histone modifications, including H3K9ac, H3K27ac, H3K4me3, and H3K9me3, at the oocyst and sporozoite stages. In this case, two replicate infections were pooled together in order to obtain enough quantity of parasite material in the mosquito for one ChIP-seq experiment. Many CVGs are upregulated during sporogonic development in the mosquito. We find that all *var* genes are silenced or expressed at low levels in the gametocyte/rings blood stages in the human host, but a single *var* gene is active during parasite development in the mosquito. The promoter region of the active *var* includes the binding site for a stage-specific AP2 transcription factor that is upregulated during sporogonic development. The active state is maintained in the infective sporozoite stage, where expression of the active *var* gene correlates with the transcription of an antisense long non-coding RNA (lncRNA).

## Results

### *P. falciparum* changes in gene expression broadly correlate with alterations in the distribution of histone modifications

Mosquitoes were infected with blood from four independent malaria-affected donors as described in the Methods section. Of these biological replicates, donor #1 carried only *P. falciparum* gametocytes, whereas the blood of donors #2, #3 and #4 carried both gametocytes and a large number of rings (Table S1). Only the gametocytes are able to infect the mosquito. In spite of the differences in the fraction of gametocytes present in the blood of the four donors, the percentage of infected mosquitoes and the mean number of oocyst found per midgut are very similar between infections (Table S1). To determine the extent of transcriptional changes that *P. falciparum* may undergo during its life cycle in the mosquito, we then carried out RNA-seq analyses on blood samples from all four human donors. We also performed RNA-seq on 7-day oocysts from the midgut of infected *An. gambiae* and 14-day sporozoites from their salivary glands obtained from infections #1 and #2. Information on the quality control steps at different points in the RNA-seq analysis is summarized in Table S2.

In order to analyze RNA-seq data (as well as ChIP-seq data described below), sequencing reads were mapped to the *P. falciparum* 3D7 genome version 25.0 (http://www.plasmodb.org). We chose this clone as reference genome based on the observation that the most prevalent *msp2* allelic family in Burkina Faso is the 3D7 type^27^, with a prevalence of 57.8% (Sondo *et al*. unpublished data). To further validate this observation, the genome of *Plasmodium* field isolates from Burkina was compared to the genome of the reference *P. falciparum* 3D7 clone as described in the Materials and Methods section. Using this approach, we could confirm that approximately 95% of all genes annotated in the 3D7 reference clone are present in the genome assembled *de novo* from Burkina samples and are uniformly distributed covering areas of high and low mappability, such as telomeric and sub-telomeric regions that contain AT-rich and repetitive sequences (Figures S1 and S2). The majority of genes present (~95%) in the *de novo* assembly have greater than 75% coverage. Only 22 genes did not have any coverage, probably because they correspond to small and repetitive sequences, such as tRNAs, and thus were discarded under our strict short-read unique mapping/scaffolding, or because the level of sequence divergence at these particular loci is higher than the number of mismatches permitted (Figure S1A). Importantly, we were able to confirm the presence of all annotated Pf3D7 CVGs, which are the least conserved genes among *P. falciparum* isolates, in the Burkina *de novo* assembly genome, including *var, rifin, stevor*, and *pfmc-2TM* (Figure S1B–D). In particular, all *var* genes were present in the *de novo* assembly with all but one having greater than 50% exonic coverage (Figure S1C,D). In spite of the similarities, the Burkina parasites contain approximately 25,000 SNPs genome-wide compared to the Pf3D7 reference strain, which is in agreement with the level of variability expected for field isolates^28^,^29^. However, these differences were not sufficiently high to affect mapping of sequencing reads of parasites present in Burkina to the 3D7 reference genome. These results justify the use of the *P. falciparum* 3D7 reference genome for the analysis of RNA-seq and ChIP-seq experiments.

**Figure 1:**
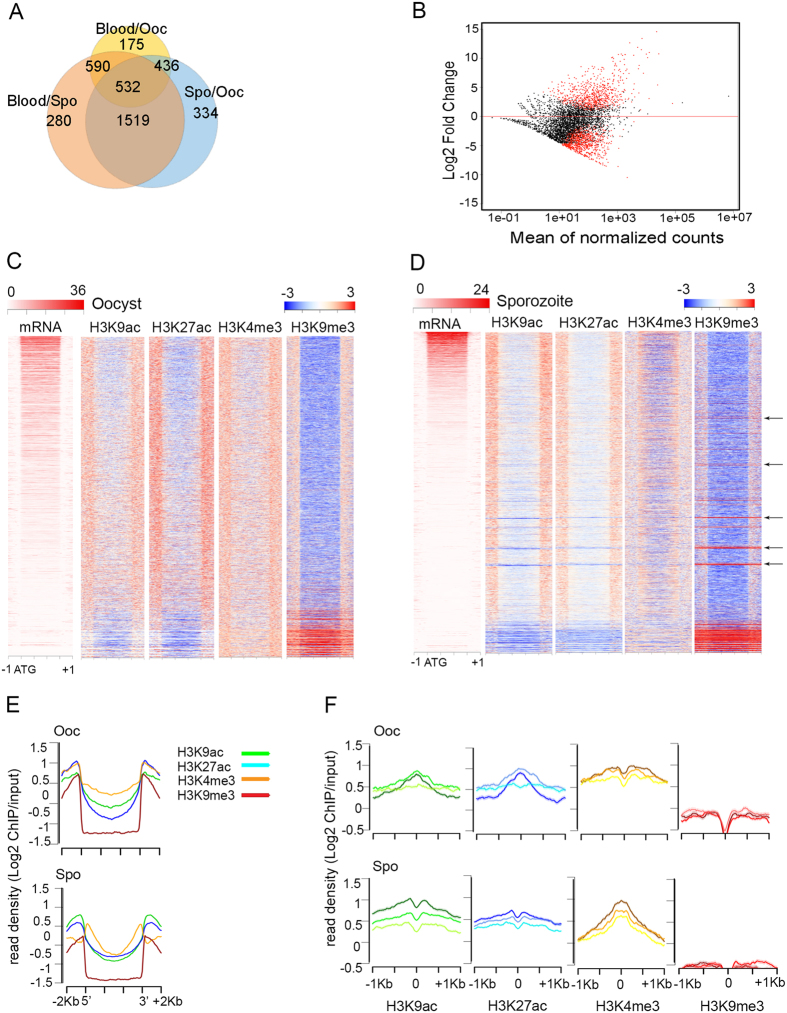
Correlation between gene expression and histone post-translational modifications during *P. falciparum* development in the mosquito. (**A**) Venn diagram showing the overlap among differentially expressed genes in various stages of the *P. falciparum* life cycle (including blood, oocyst and sporozoite stages). (**B**) MA plot where M (y-axis) is the binary logarithm of the intensity ratio and A (x-axis) is the average log intensity for a dot in the plot for differentially expressed genes between oocyst and sporozoite stages in the mosquito. Red dots indicate genes for which differences are significant for a p-value cutoff of 0.05. (**C**) Histone modification profiles in oocysts. Heatmaps correspond to ChIP-seq signal of H3K9ac, H3K27ac, H3K4me3, and H3K9me3 in oocysts ordered by RNA levels at this stage. The region comprises 1 kb upstream and downstream of the translation start and termination codons, respectively. (**D**) Histone modification profiles in sporozoites. Heatmaps correspond to ChIP-seq signal of H3K9ac, H3K27ac, H3K4me3, and H3K9me3 in sporozoites ordered by RNA levels at this stage. The region comprises 1 kb upstream and downstream of the translation start and termination codons, respectively. (**E**) Normalized/input corrected sequence reads (reads per million of reads mapped, RPKM) for each histone modification were plotted along *P. falciparum* genes comprising gene bodies and 2 kb upstream and downstream of the translation start (ATG) and termination codons, respectively. (**F**) Profile plots showing changes in levels of H3K9ac, H3K27ac, H3K4me3, and H3K9me3 in *P. falciparum* oocysts (top) and sporozoites (bottom). Genes were divided into three groups and ranked by their mRNA levels. The graphs represent normalized/input corrected ChIP-seq read unique counts (RPKM) mapped with respect to the ATG protein initiation codon for each of the three classes of genes based on RNA levels. For each graph, the darkest color represents the highest RNA levels and the lightest color represent the genes with lowest transcription.

Analysis of RNA-seq data indicates that approximately 748 genes are differentially expressed in the transition from the human gametocyte/ring stages to the oocyst, and 1317 genes between the oocyst and the sporozoite stages (FDR-corrected P-value < 0.001 and log fold change >2) (Table S3). The stage-specificity of differentially expressed genes in various stages of the *P. falciparum* life cycle is shown in Fig. 1A. Figure 1B shows the magnitude of change between the oocysts and sporozoites i.e. the log fold change as a function of mean log expression. Gene ontology analysis reveals that, compared to the sporozoite, oocyst up-regulated genes are significantly enriched in functions associated with growth, metabolism, transcription, and splicing. In contrast, the set of genes up-regulated in the sporozoite show significant functional enrichment for proteins involved in host-parasite interactions and malaria pathogenesis (Table S4).

We next carried out ChIP-seq experiments in oocysts and sporozoites on pools from two biological replicates (infections #1 and #2) using antibodies against H3K4me3, H3K9ac, H3K27ac, and H3K9me3 to examine whether changes in covalent histone modifications are associated with mosquito stage-specific transcriptional programs. Genome-wide analyses of ChIP-seq data reveal an accumulation of active histone modifications in the 5′ and 3′ regions of genes that parallels gene expression levels in oocysts (Fig. 1C) and sporozoites (Fig. 1D). A quantitative display of the location of these histone modifications with respect to gene features is shown in Fig. 1E. These patterns of occupancy during sporogonic development are consistent with data obtained from stages of the intra-erythrocytic cycle of the parasite (Figure S3)^30^.

The combined analysis of chromatin and gene expression profiles indicates a histone and stage-dependent correlation between RNA levels and the distribution and occupancy of histone modifications. In general, expressed genes show greater enrichment in active histone modifications H3K9ac, H3K27ac, and a depletion of H3K9me3, compared to silenced genes, which show the opposite pattern (Fig. 1C,D). Levels of H3K4me3 appear to stay constant irrespective of RNA levels (Kruskal-Wallis test P-value = n.s.). A quantitative analysis of the extent of association between histone modification levels and gene expression indicates a significant but weak relationship between histone enrichment and gene expression (Kruskal-Wallis test P-value < 0.0001). This association is stronger in sporozoites than in oocysts (Fig. 1F). In sporozoites, genes that are highly expressed display high levels of H3K9ac and H3K27ac compared to medium and low expressed genes. However, in oocysts, genes with intermediate expression levels have higher amounts of these histone marks than genes with either high or low expression levels (Fig. 1F). This analysis also suggests that H3K9ac and H3K27ac are better predictors of mRNA levels, compared to H3K4me3 (R squared statistics in Table S5). Statistically, levels of the three active marks combined explain approximately ~11% of the variance in gene expression levels in the sporozoite and only ~1% in the oocyst (Table S5). In the case of H3K9me3, quantitative changes in mRNA levels are not accompanied by changes in the amount of this repressive mark, and this pattern is independent of the stage of development (Fig. 1F, Table S5). For a subset of genes however, levels of H3K9me3 are high even if these genes are transcribed at medium and high levels (see arrowheads in Fig. 1D).

**Figure 2:**
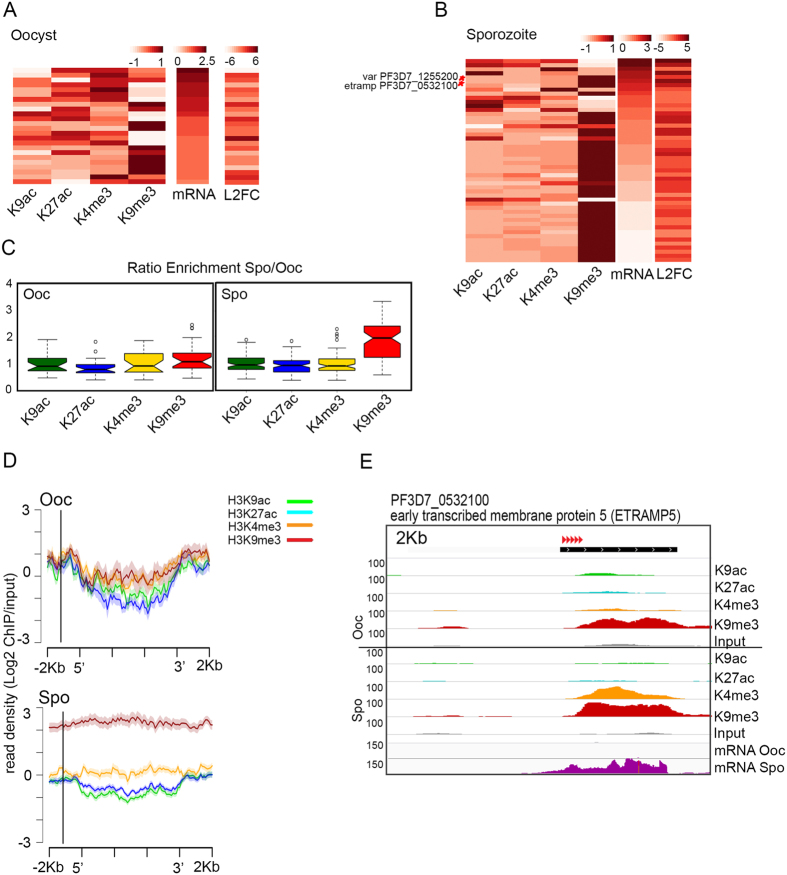
A subset of genes containing H3K9me3 are expressed during *P. falciparum* sporogonic development. (**A**) Enrichment of various histone modifications at *P. falciparum* genes (gene bodies plus 1 kb upstream of the ATG) in the oocyst displaying significant enrichment in H3K9me3 and high to medium mRNA levels. Heatmaps are sorted by levels of sense transcripts (log10 of normalized counts) in the oocyst stage. The fold change (log2) values of RNA levels for each gene in the heatmap is also depicted (L2FC < −2 are genes up-regulated in the oocyst, whereas L2FC > 2 corresponds to genes up-regulated in the sporozoite). (**B**) Enrichment of various histone modifications at genes in the sporozoite displaying significant enrichment peaks and high to medium RNA levels in gene bodies plus 1 kb upstream region. Heatmaps are sorted by RNA levels (sense) in the sporozoite stage. Labels and information are as in panel A above. (**C**) Ratio of enrichment of active and repressive histone modifications between developmental stages for oocyst (left) or sporozoite (right) genes marked with H3K9me3 and expressed at high or intermediate levels. Values above 1 indicate enrichment in the sporozoite relative to the oocyst, whereas values below 1 indicate enrichment in the oocyst relative to the sporozoite. (**D**) Density of normalized/input corrected reads for each histone modification at sporozoite or oocyst genes containing H3K9me3. The region plotted comprises gene bodies and 2 kb upstream or downstream of the translation start or termination codons, respectively. (**E**) Example of the distribution of H3K9ac (green), H3K27ac (blue), H3K4me3 (yellow) and H3K9me3 (red) profiles in the coding and flanking regions of the early transcribed membrane protein 5 (ETRAMP5) gene, which contains high levels of H3K9m3 and is differentially expressed between oocyst and sporozoite stages. Red arrows indicate direction of transcription. The ChIP-seq signal corresponds to the same number of reads between the two parasite stages. The input control is also displayed and all tracks are show at equal scale.

To examine the relationship between H3K9me3 levels and transcription in more detail we selected genes that show significant H3K9me3 enrichment (MACS peaks intersecting gene bodies and/or the 1 kb region upstream of the ATG), while displaying high to medium levels of gene expression in either the oocyst or sporozoite stages. A total of 24 genes in the oocyst and 74 genes in the sporozoite fit this criterion. We then compared transcript levels with the presence of various histone modifications. For oocyst genes containing H3K9me3, higher levels of this modification correlate with lower levels of sense mRNAs, although genes containing high levels of this modification are still expressed at intermediate levels (Fig. 2A). The same relationship can be observed in the sporozoite. However, a number of genes containing very high levels of H3K9me3 are highly expressed at this stage (Fig. 2B). Interestingly, many of these H3K9me3-containing genes correspond to developmentally regulated genes that show their peak of expression in the sporozoite (42 differentially expressed genes, FDR-corrected P-value < 0.001 and log fold change >2, Table S6). These genes do not lose this mark upon activation and, instead, H3K9me3 levels rise when their transcription increases. This is clearly shown when examining the ratio of enrichment of H3K9me3 in the sporozoite relative to the oocyst, for oocyst expressed genes (left panel) or sporozoite expressed genes (right panel), which is above 1 for sporozoite up-regulated genes (Fig. 2C). Likewise, the results also show a 2-fold enrichment in H3K9me3 in sporozoite vs. oocyst up-regulated genes (Fig. 2D). These results suggest that, during sporozoite stages, a subset of genes are actively transcribed in spite of the presence of high levels of H3K9me3. Gene ontology analyses on the set of H3K9me3 enrichment peaks that intersect genes that are otherwise expressed reveals that H3K9me3 localizes near or at genes encoding for proteins involved in interactions with the host, including CVGs in telomeric and sub-telomeric regions (Table S6). These genes are not necessarily located in close proximity, suggesting that they are not under coordinated regulation. An example of the chromatin profile of one of these genes expressed in the sporozoite but enriched in H3K9me3, which belongs to the ETRAMP (early transcribed membrane proteins) gene family of virulence factors, is shown in Fig. 2E.

### Transcription of clonally variant gene families during *P. falciparum* sporogonic cycle in mosquitoes

**Figure 3:**
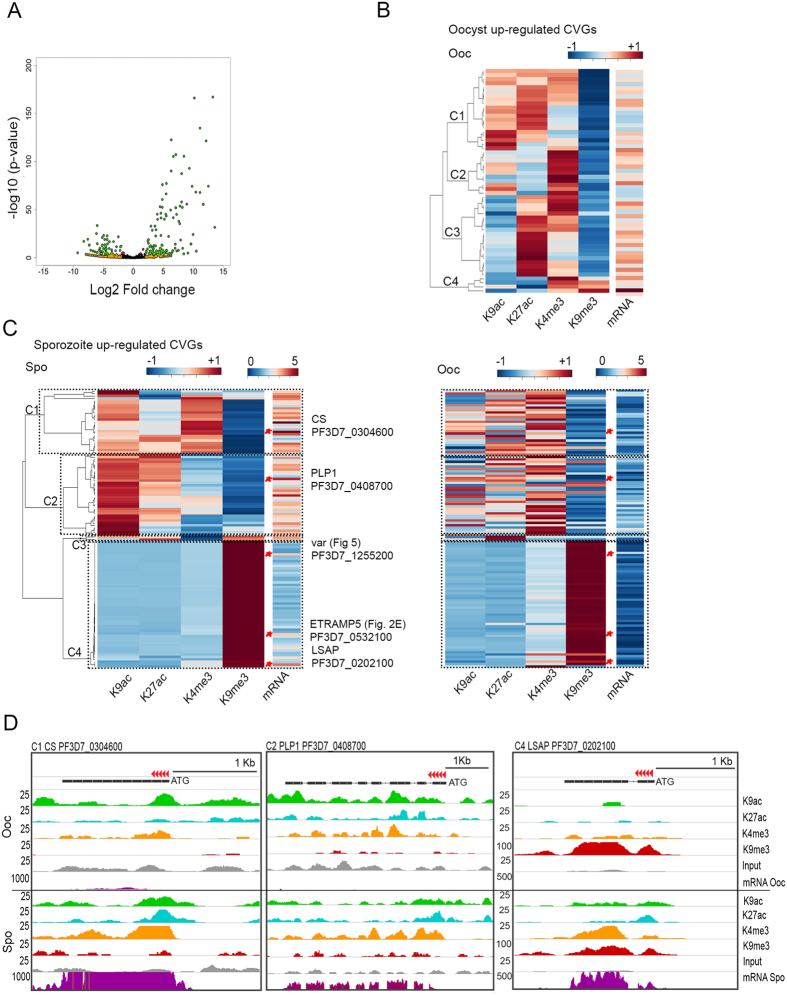
Histone modifications and transcription in developmentally regulated clonally variant genes. (**A**) Volcano plot of clonally variant gene families showing differential gene expression during the oocyst-sporozoite stage transition. Points are colored according to the magnitude of change and statistical significance (Orange: Log2 Fold Change >2 or <−2; Red: P < 0.001; Green: Log2 Fold Change >2 or <−2 and P < 0.001; and Black: none of the above). (**B**) Dendogram showing levels of enrichment in active and repressive histone modifications (H3K9ac, H3K27ac, H3K4me3 and H3K9me3) and RNA levels for CVGs expressed in oocysts but not in sporozoites (oocyst-specific CVGs). Only those genes with FDR-corrected P-value < 0.001 and >2.0 fold change are considered. Enrichment are log10 transformed values. The region considered to calculate the average enrichment comprises gene bodies and 1 kb upstream of the translation start codon. (**C**) Dendogram showing levels of enrichment in active and repressive histone modifications (H3K9ac, H3K27ac, H3K4me3 and H3K9me3) and RNA levels for sporozoite-specific CVGs (left panel). Only those genes with FDR-corrected P-value < 0.001 and >2.0 fold change are considered. Enrichment are log10 transformed values. The region considered to calculate the average enrichment comprises gene bodies and 1 kb upstream of the translation start codon. The right panel shows chromatin states and RNA log2 fold change expression values in the oocyst stage for the set of sporozoite-specific CVGs, sorted by the same gene order as in the dendogram on the left. The comparison shows changes associated with the activation of these genes prior to transmission (not shown in panel C). Asterisks point to examples of genes in panel D. (**D**) Examples of CVGs showing various combinations of histone modifications. Genes representative of each cluster found in panel C are highlighted. Tracks for H3K9ac (green), H3K27ac (blue), H3K4me3 (yellow) and H3K9me3 (red) correspond to the same number of reads between the two parasite stages. Red arrows indicate direction of transcription.

Previous studies in rodent malaria suggest that transmission through the mosquito host can affect parasite virulence by altering the expression profiles of multigene families involved in pathogenesis^24^, but results reported by other studies in rodent *Plasmodium* and different vector species are controversial^26^. Whether similar changes in CVGs expression occur in *P. falciparum* during its sporogonic development in *An. gambiae* has not been investigated in detail, and the mechanisms underlying these changes remain unknown. Here we follow the definition of CVGs established by Rovira-Graells *et al*.^15^. These include genes involved not only in immune evasion (i.e. *var, rifin* and *stevor*), but also genes linked to lipid metabolism, protein folding, erythrocyte remodeling, or transcriptional regulation. Experiments described above suggest that some CVGs are present among the group of transcribed genes containing H3K9me3. To gain further insights into the mechanisms of CVG transcription regulation in mosquitoes, we first examined stage-specific changes in gene expression that occur in *P. falciparum* CVG families during the developmental transitions from the oocyst to sporozoite stages. The results show that most CVGs are developmentally regulated in the mosquito, with 55 CVGs expressed in the oocyst and 118 in the sporozoite (Fig. 3A, Table S7). Proteins encoded by sporozoite up-regulated CVGs include malaria vaccine candidates such as the circumsporozoite (CS) protein (PlasmoDB Gene ID PF3D7_0304600, TRAP-like family), liver stage antigen 3 (LSA3) (PF3D7_0202100, PHISTb protein sub-family), reticulocyte binding protein (PF3D7_0402300, PfRh), and one *rhoph1*/*clag3* gene (PF3D7_0302200), among others (Table S7, Rovira-Graells *et al*.^15^). However, except for *var* genes (see below), other well-known clonally variant gene families like *rif, stevor*, and *Pfmc-2TM*, are not expressed or expressed at very low levels in the oocyst or sporozoite stages. Only one gene, *surf8.2*, is differentially regulated with a peak of expression in the oocyst stage (Table S7).

To test whether covalent histone modifications correlate with the changes in expression of CVGs during *P. falciparum* sporogonic development, we first identified CVGs that are upregulated in the sporozoite with respect to the oocyst and used the ChIP-seq signal for H3K9ac, H3K27ac, H3K4me3 and H3K9me3 to carry out hierarchical clustering. The results suggest that many of the developmentally regulated CVG families show a mixed pattern of enrichment in active and repressive histone modifications. Oocyst up-regulated CVGs, i.e. genes that are expressed at higher levels in the oocyst (log2 Fold Change <−2, FDR-corrected P-value < 0.001), are all depleted in H3K9me3 (Fig. 3B). Various groups of genes can be identified based on levels of enrichment in active marks. Genes in cluster 1 contain both H3K9ac and H3K27ac at medium levels, and a subset of them also contain H3K4me3. Cluster 2 includes genes that are marked with high amounts of H3K4me3, whereas genes in cluster 3 are enriched in H3K27ac and H3K4me3 (Fig. 3B). These results suggest that *P. falciparum* may use different combinations of active histone modifications during the transcription of CVGs in the oocyst stage.

In the sporozoite up-regulated CVGs, i.e. genes that display higher mRNA levels in the sporozoite than in the oocyst (log2 Fold Change >2, FDR-corrected P-value < 0.001), four different gene cluster combinations were also identified based on their histone modification profiles (Fig. 3C, left panel). In order to compare changes in transcription and histone modifications for these genes in the transition from oocyst to sporozoite, we used the clustering matrix obtained in sporozoites to visualize ChIP-seq and RNA-seq data obtained in oocysts (Fig. 3C, right panel). In cluster C1, the active state is linked to an enrichment of H3K9ac and H3K4me3 and a depletion of H3K27ac and H3K9me3. These genes gain H3K9ac and H3K4me3 in the developmental transition from oocyst to sporozoite. Cluster 2 corresponds to genes containing high levels of H3K9ac and H3K27ac active modifications and depletion of H3K9me3. These genes gain H3K9ac and H3K27ac in the transition from the oocyst to the sporozoite and mostly correspond to genes expressed at intermediate levels (Fig. 3B). The third cluster includes two genes that become enriched in both H3K9ac and H3K9me3 whereas levels of H3K4me3 decrease. Genes in clusters 1–3 fall in the category of high/medium expressed genes. Cluster 4 genes display very high levels of H3K9me3 and are generally depleted of active histone modifications, both in the oocyst and sporozoite, and correspond to genes that are generally expressed at medium/low levels in the sporozoite (Fig. 3B, Table S7). Examples of histone modification and mRNA profiles for genes representative of clusters 1, 2 and 4 that encode proteins with known functions in host cell invasion, such as circumsporozoite protein (CS), perforin-like protein 1 (PLP1) and liver stage associated protein (LSAP), are shown in Fig. 3D. These results suggest that the regulation of expression of CVGs in the mosquito during the transition from the oocyst to the sporozoite involves increased accumulation of different active histone modifications, mostly in the 5′ region of the gene (see examples for CS and PLP1 in Fig. 3D). However, activation of genes containing H3K9me3 in the oocyst appears to primarily involve a decrease in H3K9me3 throughout the gene (LSAP in Fig. 3D).

### AP2 transcription factors may play a role in *P. falciparum* phenotypic plasticity in the mosquito

**Figure 4:**
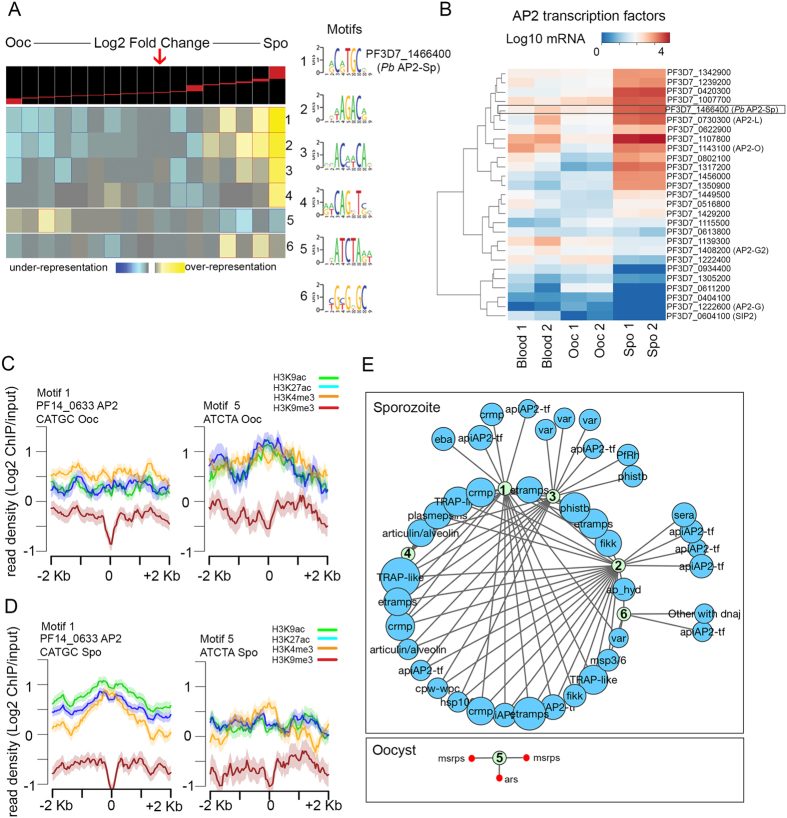
AP2 transcription factors are developmentally regulated and their binding sites are present in CVGs. (**A**) Motif analysis using FIRE. Each row corresponds to a predicted motif and columns are groups of genes with a similar expression value (log2 ratio). The heatmap indicates whether each motif is overrepresented (yellow) or underrepresented (blue) in each expression level gene cluster. The panel at the top is the histogram of the fold change (log2) values observed in the set of differentially expressed genes between oocyst and sporozoite stages. The arrow indicates the threshold log2 value that separates oocyst and sporozoite specific genes. (**B**) Dendogram of mRNA levels of the AP2 family of transcription factors in the blood of human donors #1 and #2 and in the oocyst and sporozoite stages from these two infections. The PF3D7_1466400 AP2 transcription factor with binding sites in the promoter sequences of sporozoite-specific genes based on the FIRE analysis is highlighted. (**C**) ChIP-seq density (rpm/input) plots of H3K9ac, H3K4me3, H3K27ac, and H3K9me3 present during the oocyst and sporozoite stages in the region surrounding the binding motifs identified by FIRE and present in target genes. (**D**) Cytoscape network showing predicted regulatory interactions between binding motifs identified by FIRE and their target genes. Sporozoite up-regulated genes are colored in blue (upper panel) and those up-regulated in the oocyst are in red (lower panel). The size of the circles is proportional to the magnitude of the differential expression between stages (log2 fold change).

Transcriptional regulation of CVGs during the oocyst to sporozoite transition may be the result of the binding of transcription factors to the regulatory regions of genes. Transcription factors can participate in gene regulation by recruiting components of the transcription complex or protein complexes involved in posttranslational histone modifications and nucleosome remodeling^31^. Interestingly, *P. falciparum* genes we find to be developmentally regulated in the mosquito include the Api-AP2 family. This family of DNA-binding proteins is the sole group of candidate transcription factors in *Apicomplexa*, and they are largely conserved across *Plasmodium* species^32^. To date, 27 of these transcription factors have been described in *P. falciparum*^33^,^34^ but only two have been reported to be associated with gene expression in the mosquito life cycle stages in rodent *P. berghei* malaria parasites^13^,^35^,^36^. To explore the possibility that these factors play a regulatory role in stage-specific alterations in gene expression and chromatin structure of CVGs we identified putative binding sites for AP2 transcription factors on differentially expressed genes during the mosquito life cycle. We used the Finding Informative Regulatory Elements (FIRE) algorithm, which searches for motifs whose presence or absence is highly informative of the associated expression values^37^. This analysis reveals that sporozoite-specific genes are significantly enriched in binding sites for various motifs. Of the six motifs reported, three are linked to the regulation of genes specific to the sporozoite stage, whereas one is associated with genes expressed in the oocyst stage (Fig. 4A). The motif with the highest significance score, motif 1 [AG]C[AG]TGC[AGT], is most frequently found in the upstream regions of sporozoite-specific genes and is identical to the recognition sequence of the AP2 transcription factor PF3D7_1466400. The orthologous gene in *P. berguei* (AP2-Sp, PBANKA_1329800) has been previously shown to be essential for the formation of sporozoites^33^,^34^,^35^,^36^. Differential gene expression analysis of RNA-seq data for this family of transcription factors indicates that PF3D7_1466400 is developmentally regulated during the gametocyte/trophozoite stages and life cycle in the mosquito, and that the RNA for this gene reaches its peak of expression in sporozoites (Fig. 4B, Table S3). The orthologous genes of other AP2 transcription factors previously characterized in *P. berguei* that have functions in the regulation of gene expression in the mosquito stages, named AP2-O, and AP2-L (PF3D7_1143100 and PF3D7_0730300 in *P. falciparum*), are also developmentally regulated in *P. falciparum* during the lifecycle in the mosquito, and reach their peak of expression in the sporozoite stage (Fig. 4B).

To examine the possible involvement of PF3D7_1466400 AP2 in the regulation of transcription during sporogonic development, we then carried out a detailed analysis of the occurrence of histone modifications in the target genes predicted by the FIRE analysis to have the binding motif for this AP2 transcription factor. Results from this analysis suggest stage-specific changes in chromatin structure at these sites. That is, when plotting the average profiles of histone modifications for target genes containing the [AG]C[AG]TGC[AGT] motif in the promoter region, we find higher levels of active histone modifications in sporozoites compared to the same set of genes in the oocyst stage, when these genes are silent (Fig. 4C, Table S8). On the other hand, the set of target genes containing motif 5 [AGT]ATCTA[AG][AT] (Fig. 4A), which is overrepresented in oocyst-specific genes, shows the opposite pattern, with greater enrichment of active marks in the oocyst stage compared to the sporozoite (Fig. 4D).

The PF3D7_1466400 binding site motif is present in 126 sporozoite-specific genes, of which 21 are developmentally regulated CVGs. These include a number of *var* genes, Clag3.1, Centrin 3, ETRAMPS, calcium dependent protein kinases (CAMK), and Sir2A (Table S8). Network analysis of the relationship between transcription factor motif sites and the predicted target genes belonging to CVG families suggests a highly connected network (Fig. 4E). Importantly, several genes appear to be co-regulated based on the number of interconnected nodes in the network, thus the same target gene can have binding sites for more than one transcription factor (Table S8). In addition, among these targets are genes encoding AP2 transcription factors, which are also up-regulated in the sporozoite, pointing to the existence of positive feedback loops for regulation of these factors (Fig. 4E, Table S8). Only three CVGs are among the target genes predicted in the oocyst stage to have binding sites for the set of motifs identified by the FIRE analysis (bottom box in panel 4E). These results support the idea that AP2 transcription factors have roles in the regulation of stage-specific genes involved in immune evasion and in preparing the parasite for transmission and human host cell invasion.

### Expression of *var* genes during the sporogonic cycle

Among the set of CVGs developmentally regulated in the mosquito we identified genes belonging to the *var* family. *P. falciparum var* genes encode malaria virulence factor Erythrocyte Membrane Protein 1 (PfEMP1). Epigenetic mechanisms involving histone modifications, nuclear positioning, and lncRNAs, have been suggested to be involved in the control of *var* gene transcription during the blood stages of the parasite^19^,^20^,^21^,^22^,^24^,^38^. However, how *var* gene expression is regulated during the sporogonic development in mosquitoes is not understood.

**Figure 5:**
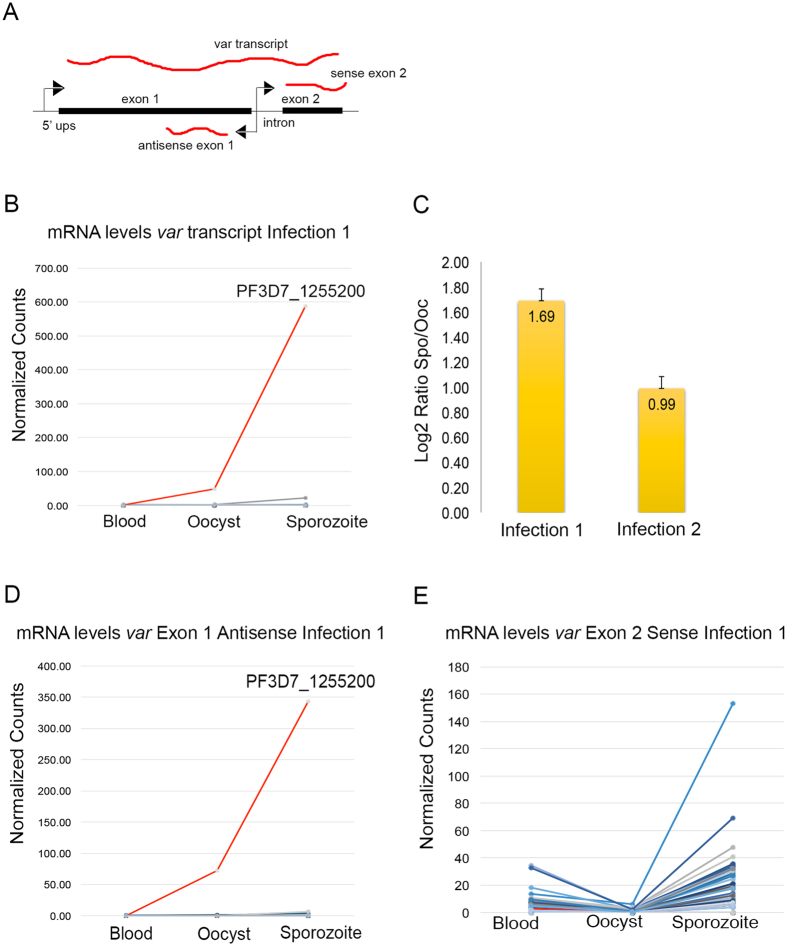
Gene expression patterns of *var* genes in the blood of human donors and during the sporogonic development of *P. falciparum* after infection. (**A**) Schematic map of a typical *var* locus showing the different types of RNAs transcribed from the 5′ upstream region and the intron. (**B**) Changes in the expression of the full-length sense transcript for all *var* genes during the blood, oocyst and sporozoite stages. The figure shows the results of the infection for donor #1, which had only gametocytes present in the blood. Only the RNA corresponding to the active PF3D7_1255200 *var* gene is visible (red), since the rest of the *var* genes express no or very low levels of this transcript. (**C**) Results of the qRT-PCR assay for the active PF3D7_1255200 *var* gene using primers targeting a region in exon 1. The results suggest upregulation of this *var* gene during the oocyst to the sporozoite transition in two different experimental infections. (**D**) Expression of exon 1 antisense lncRNAs for all *var* genes in the blood, oocyst and sporozoite stages during infection #1. Only the transcript corresponding to the PF3D7_1255200 *var* gene is visible (red). (**E**) Expression of exon 2 sense RNAs for all *var* genes in the blood, oocyst and sporozoite stages during infection #1. In this case, all *var* genes express this transcript, which is thought to be involved in *var* gene silencing. Levels of this RNA corresponding to the PF3D7_1255200 *var* gene (red) are low.

During the intra-erythrocytic life cycle, active *var* genes express a full-length sense transcript that encodes the PfEMP1 protein. In addition, *var* genes encode an exon 2-specific sense RNA transcribed from a bidirectional intronic promoter that has been suggested to repress *var* gene expression^24^. Finally, the same intronic bidirectional promoter transcribes an antisense exon 1 lncRNA, which has been previously shown to be involved in the activation of *var* gene expression^24^ (Fig. 5A). Results from the RNA-seq analysis show differences in mRNA levels of *var* genes between gametocyte/ring and mosquito *P. falciparum* stages. All blood donors analyzed carry a mixture of gametocytes and rings, except for donor #1, whose blood only contains gametocytes. We observed no expression of any *var* gene in the blood of this donor prior to mosquito infection (Fig. 5B). The blood samples of donors # 2 and # 3, which carry a mixed population of rings and gametocytes, display medium to low levels of expression of most *var* genes (Figure S4A). These observations suggest that *var* genes are not transcribed in gametocytes, and that the observed expression in donors #2 and #3 arises from the trophozoites present in these samples. After infection of the mosquito host and gamete fusion, there is no *var* gene expression detected in *P. falciparum* oocysts, except for PF3D7_1255200, which transcribes the full-length sense mRNA at this stage (Fig. 5B). This same *var* gene undergoes a dramatic 6-fold increase in mRNA levels in the transition to the sporozoite (Fig. 5B, S4A). These changes in expression were validated by qRT-PCR (Fig. 5C) and are consistent across infections 1, 2, and 3 (Fig. 5B, S4A). It is interesting to note that the same *var* gene is predominantly expressed in all three experimental infections in *An. gambiae* (Fig. 5B, Figure S4A). This is independent of the human donor and in spite of the heterogeneity of the parasite population in the human host, where multiple *P. falciparum* clones often co-occur. Finally, this observation is unlikely to be due to technical issues, such as differential mappability of RNA-seq reads to different *var* genes, since ChIP-seq reads for various histone modifications map uniquely to the different *var* genes.

The expression of only one *var* gene in the sporozoite that is the same in all different infection experiments suggests a selection process, either because this specific PfEMP1 protein plays a unique role in the life cycle of the parasite in the mosquito or because this protein does not have deleterious effects on the host. To distinguish between these possibilities, we carried out an additional infection experiment using blood from donor #4 and *An. coluzzii* instead of *An. gambiae*. These two species correspond to the former R and S forms of *An. gambiae* that have been recently separated into different species^39^. *An. coluzzii* and *An. gambiae* are both natural malaria vectors in the study area of Burkina Faso but they differ in their susceptibility to infection^40^. Consequently, if there is selection by the mosquito immune system for the expression of a specific *var* gene we may expect a differential response between the two species in terms of *var* gene expression. RNA-seq experiments show that the same PF3D7_1255200 *var* gene is expressed in *An. coluzzi*, although in this case expression is highest in the oocyst stage (Figure S4A). These results suggest that selection for the expression of a unique *var* gene is independent of the differences between *An. gambiae* and *An. coluzzi*.

To gain insights into the mechanisms by which *var* genes are differentially transcribed in the oocyst to sporozoite transition, and the observation that a specific *var* gene is turned on during the oocyst stage i.e. mutually exclusive expression, we examined the distribution of histone modifications in the promoter region of *var* genes. PF3D7_1255200 belongs to the UPSB class of *var* genes, whose promoters are located around 50 bp upstream of the ATG^41^. The *in silico* analysis performed by Campbell *et al*.^33^ revealed binding sites for the PF3D7_1466400 3 (*P. berghei* AP2-Sp) transcription factor ([AG]C[AG]TGC[AGT]) in a number of UPSB *var* genes. This corresponds to the motif we find over-represented in sporozoite-expressed genes (Fig. 4A). Using FIMO motif scan analysis, 7 *var* genes were identified as containing the PF3D7_1466400 binding motif in the promoter region (±150 bp of the ATG) (p < 0.001, E-value < 10), including the active *var* gene PF3D7_1255200 (Table S9). We then examined chromatin signatures in the promoter region of these subset of genes and found that the active *var* gene shows lower levels of H3K9me3 compared to other *var* genes that also contain the PF3D7_1466400 motif (Figure S5).

Long non-coding RNAs have been proposed to regulate *var* gene activation during the intra erythrocytic cycle *in vitro*^24^. Thus, in addition to AP2 transcription factors and histone post-translational modifications, we examined expression of the sense exon 2 and antisense exon 1 transcripts. The lncRNA antisense transcript corresponding to exon 1 of the active PF3D7_1255200 *var* gene displays a pattern of expression similar to that of the full sense transcript. On the other hand, all silenced *var* genes express the antisense lncRNA at negligible (infection 1 and 4) or low (infections 2 and 3) levels (Figs 5D and S4A). Interestingly, for infection 2, the second highest antisense lncRNA level corresponds to PF3D7_0533100, which is a pseudogene that does not encode a functional protein. These observations agree with a model whereby expression of the antisense lncRNA results in activation of the sense full-length mRNA of the active PF3D7_1255200 *var* gene.

## Discussion

The molecular mechanisms underlying changes in gene expression in *P. falciparum* during sporogonic development have not been explored in detail. Here we examine changes in the transcriptome of *P. falciparum* after infection of *An. gambiae* and we correlate these changes with alterations in histone modifications, expression of AP2 transcription factors, and levels of various lncRNAs. Previous work suggests dynamic changes in the occupancy patterns of histone modifications during the intra-erythrocytic life cycle of *P. falciparum*^42^, as well as an association between some active histone modifications and gene expression^30^,^43^. Similarly, during *Plasmodium* development in the mosquito, H3K9ac and H3K27ac enrichment, but not H3K4me3, show a significant but weak correlation with increased levels of gene expression in sporozoites. However, levels of all active histone modifications are uncoupled from RNA levels in oocysts. These differences could be related to the oocyst-specific transcriptional program, which includes expression of genes involved in housekeeping functions.

The pattern of distribution and occupancy of the repressive H3K9me3 histone modification at silenced loci agrees with previous studies in the blood stages of the parasite^19^,^44^. However, we find a set of high and medium expressed genes that are marked with high levels of H3K9me3, some of which correspond to developmentally regulated genes that show both active and repressive histone modifications. Genes for which we observed such a bivalent state, presence of H3K9ac and/or H3K4me3 and enrichment in H3K9me3, participate in diverse processes such as cellular metabolism and transport (*acs, acbp*, lysophospholipases), in protein folding/stability (*phistb*/*dnaj*), or in erythrocyte invasion (*eba, etramp* and *var*), among other functions. About half of H3K9me3 containing genes belong to clonally variant gene families. Since we are examining natural isolates of *P. falciparum*, it is possible that the pattern we observe results from a mixed infection of parasite clones that display heterogeneous expression and/or epigenetic profiles. However, this hypothesis does not explain the switch in expression of most genes that we observe between the oocyst and the sporozoite stages in the mosquito. Alternatively, the presence of H3K9me3 at expressed genes agrees with recent studies that have reported the presence of this histone modification throughout the coding region of active *Drosophila* and mammalian developmentally regulated genes^45^,^46^. This pattern has been associated with activation of gene expression by signaling the recruitment of RNA polymerase II^45^. H3K9me3 also appears to participate in alternative splicing^46^. This bivalent chromatin state is also reminiscent of that described for stem cells, in which genes that will become activated or repressed during the establishment of different cell lineages display a combination of active and repressive histone marks^47^,^48^. Like stem cells, the *Plasmodium* life cycle relies on two seemingly opposite requirements: to keep genes off at a specific stage while retaining the ability to switch them on quickly when needed^49^. The priming of genes involved in malaria pathogenesis in humans at the sporozoite stage is in line with previous proteomic and transcriptomic studies^3^,^50^,^51^,^52^,^53^,^54^. Compared to these studies, our system is unique in that it mimics transmission conditions in nature, and therefore adds important evidence to support the idea that the passage of *P. falciparum* through the mosquito can affect parasite virulence at a later infection^25^,^55^.

Only one *var* gene is transcribed in ring stages during the life-cycle of *P. falciparum* in the human host whereas all other *var* genes are silenced^18^,^56^. However, little is known about *var* gene regulation in other stages of the parasite life-cycle. In addition, an important unresolved question in the field is whether the regulation of *var* genes during sporogonic development of *P. falciparum* affects malaria virulence. A recent study examined the dynamics of *var* gene expression in patients infected with mosquito-passaged *P. falciparum* parasites via quantitative real-time PCR and reported changes in *var* gene expression between a parental parasite culture line, and the parasites isolated from the infected volunteers^57^. The authors suggest that this may be the result of epigenetic reprogramming in the mosquito prior to infection of the human, but no data is presented supporting this suggestion. Here, we report for the first time the transcriptional dynamics of these genes during parasite development in the mosquito and show that *P. falciparum* maintains all *var* genes in a transcriptionally silent state with the exception of one that is active in the midgut oocysts, and reaches its peak of expression in the salivary gland sporozoites prior to transmission. When dealing with natural infections, blood from each infected donor may contain a mixed population of different parasite clones. Despite this, results indicate that the active *var* gene is the same in all four experimental infections performed. Since PfEMP1 is a parasitized erythrocyte protein that does not have *a priori* a function in the free-living stages of the parasite in the mosquito, it is possible that what we detect corresponds to cryptic *var* gene transcription. These nascent mRNAs could also have a regulatory role in the monoallelic expression of this family^23^. The question of the functional properties of these transcripts in the mosquito remains an issue for future investigation.

Expression of *var* genes during the erythrocytic life cycle has been linked to changes in the levels of histone modifications^19^,^20^,^21^,^38^. In this study, however, there is no causal link between enrichment in active/repressive histone modifications and the switching and activation at *var* genes, suggesting that the role of histone modifications might be secondary to other regulatory events^8^. Accumulating evidence suggests that AP2 transcription factors play a critical role in gene regulation in *Plasmodium*^32^. Previous studies identified binding sites for various AP2 transcription factors, originally named SPE1, CPE, and SPE2, that are involved in *var* gene silencing^58^,^59^. In particular, the SPE1 regulatory sequence matches the binding site motif for the AP2 transcription factor PF3D7_1466400^33^. The expression of the PF3D7_1466400 gene in *P. falciparum* asexual stages has been confirmed by microarrays and mass-spectrometry-based proteomic approaches^34^. During the sporogonic cycle in the mosquito, the same AP2 transcription factor probably initiates the expression cascade of sporozoite specific genes by binding directly to a cis-acting control element [AG]C[AG]TGC[AGT] in their 5′ upstream regions. Therefore, binding of PF3D7_1466400 to the promoter region of the active PF3D7_1255200 *var* gene likely contributes to its activation. However, results from previous studies indicate the expression of this transcription factor in various blood stages of the parasite in the human host, which argues against the sporozoite specific expression of this transcription factor, even if experimental evidence from the orthologous gene in *P. berghei* supports this conclusion^35^. It is thus possible that various AP2 factors act cooperatively in activating the sporozoite specific transcriptional cascade in *P. falciparum*, as shown in our network analysis and recently proposed by others^60^. Altogether, our results lead us to speculate that the PF3D7_1466400 AP2 transcription factor, which reaches its maximum expression at the sporozoite stage in the mosquito, is recruited to the PF3D7_1255200 gene. The binding of this and possibly additional AP2 transcription factors would then turn on the expression of the full-length sense transcript and the exon 1 antisense lncRNA.

Various alternative hypotheses can be put forward to explain the biological significance of the expression of a single *var* gene during *P. falciparum* development in the mosquito host. One possibility is that the active PF3D7_1255200 *var* gene is being pre-selected, by a parasite-driven mechanism, for later infection of the human host. This hypothesis is based on the assumption that there is a predominant parasite clone that is best adapted to the human host population in Burkina Faso. If this were the case, we would expect the same *var* gene expressed in the mosquito to be active in the ring stage of the parasite in the human host. Results for the RNA-seq experiments suggest that multiple *var* genes, but not Pf3D7_1255200, are expressed in the blood of donors containing a mixture of rings and gametocytes. RNA expression data for the early blood stages suggest the active *var* gene has been selected *de novo* in the mosquito (i.e. resetting), rather than being previously transcribed in ring stages (i.e. memory). An alternative explanation to selection by the human host is that the PfEMP1 variant encoded by the active PF3D7_1255200 *var* gene plays a specific function during *Plasmodium* development in the mosquito host. For example, one can speculate that this PfEMP1 variant participates as a ligand in mediating adhesion of the ookinete to the midgut epithelium as it has been described for ICAM1, CD36, CR1, and CD31 human host cell receptors^61^. Finally, the fact that we observe the same *var* gene expressed in each experimental infection in mosquitoes is similar to the case described for pregnant women. In this case the same *var* gene coding for VAR2CSA, which adheres to a specific host cell receptor named chondroitin sulphate A, is selected in the course of a placental infection^62^. In the context of the natural conditions of transmission in Burkina, the interaction between *P. falciparum* field isolates with the local mosquito populations could have favored selection of this particular *var* gene if there is a survival advantage to the mosquito by conferring or increasing resistance to disease. Other types of selection events related to pleiotropy or gene linkage of the selected variant to certain drug-resistance alleles cannot be ruled out^63^.

Our findings provide insights to explain the regulation of clonally variant genes during parasite development in the mosquito. In this model, regulation of *var* gene expression involves resetting and *de novo* activation of a single variant in the mosquito parasite stages. That is, since the same *var* gene is silent in the gametocyte/ring blood stages, this finding is most likely explained by an epigenetic resetting of the human-acquired *var* gene repertoire. The fact that mutually exclusive expression takes place during parasite development in the mosquito is significant, and opens new and exciting opportunities to study the mechanisms of *var* gene regulation *in vivo*. With respect to the biological basis of this pattern, this new variant may be required for parasite life cycle progression and adaptation to the mosquito host. More generally, our results add new insights into the role of epigenetic processes in the mosquito stages of the human malaria parasite, which until now remained uninvestigated. Understanding of the processes controlling sporogonic development is crucial, since mosquitoes are at the center of malaria control interventions.

## Materials and Methods

### Mosquito rearing and dissection

Three- to five-day-old female *An. gambiae* mosquitoes were sourced from an outbred colony established in 2008 and repeatedly replenished with F1 from wild-caught mosquito females collected in Kou Valley (11°23′14″N, 4°24′42″W), 30 km from Bobo-Dioulasso, south-western Burkina Faso (West Africa). Mosquitoes were maintained under standard insectary conditions (27 ± 2 °C, 70 ± 5% relative humidity, 12:12 LD). Four independent experimental infections, biological replicates, were carried out by membrane blood feeding in the laboratory as described previously^64^,^65^,^66^,^67^. Carriers, gametocytemia, number of mosquitoes infected and analyzed, and prevalence and intensity of infection at the oocyst stage are included in Table S1. Briefly, females were fed through membranes on gametocyte-infected blood from malaria patients. Venous blood was collected and the serum was replaced by a non-immune AB serum to avoid transmission of human blocking factors. Dissection of mosquito midguts and salivary glands was performed *in situ* on adult females at 7 (oocyst) and 14 days (sporozoite) post-blood meal through sporogonic development. Tissues were maintained in ice-cold Schneider’s insect culture medium (Sigma-Aldrich) and fresh tissues were immediately processed for chromatin and RNA analyses.

### Chromatin immunoprecipitation and sequencing

Chromatin immunoprecipitation was performed as described previously^68^. Antibodies to histone modifications used in this study were anti-H3K9ac (Millipore #07–352), anti-H3K4me3 (Abcam ab8580), anti-H3K27ac (Abcam ab4729), and anti-H3K9me3 (Abcam ab8898). These are ChIP-grade antibodies and have been previously assayed in *Plasmodium*^30^,^69^,^70^. ChIP-seq libraries were prepared following a protocol optimized for low DNA quantities^71^. Due to the difficulties in obtaining enough parasite DNA from mosquito tissues, two biological replicates for each time point were pooled together prior to chromatin immunoprecipitation. ChIP-seq libraries were amplified using the HiFi Kapa Sybr library preparation kit (KapaBiosystems). Use of this enzyme eliminates mapping biases due to the high AT content of the *Plasmodium* genome^72^,^73^. ChIP-seq libraries were sequenced at the HudsonAlpha Institute for Biotechnology using an Illumina HiSeq2000 sequencer.

### RNA isolation and sequencing

RNA-seq was used to generate comprehensive transcriptome profiles of *P. falciparum* during its sporogonic development at the gametocyte/ring, the oocyst, and the sporozoite stages. Two to four biological replicates per stage were analyzed. Total RNA was extracted from whole blood, fresh midguts and salivary glands using the mirVana RNA Isolation Kit (Ambion) according to the manufacturer’s protocol. RNA concentration was quantified using a Qubit 2.0 Fluorometer and RNA integrity was determined with an Agilent 2100 Bioanalyzer. After ribosomal reduction (RiboMinus Eukaryote Kit, Ambion), standard directional RNA-seq libraries were prepared and sequenced at the HudsonAlpha Institute for Biotechnology using an Illumina HiSeq2000 sequencer.

### Quality control, reference genome and assembly

Quality analysis of Illumina reads was performed using FastQC (http://www.bioinformatics.bbsrc.ac.uk/projects/fastqc) (Table S2). Reads with a quality score under 20 were discarded. The alignment and accuracy statistics were computed using QualiMap v2.1.3^74^ (Table S2).

All sequencing reads were mapped to the *P. falciparum* 3D7 genome version 25.0 (http://www.plasmodb.org). To validate this choice, the *Plasmodium* field isolates from Burkina were compared for similarities to the assembled 3D7 *P. falciparum* reference genome. ChIP and Input reads were first filtered by removing all sequences mapping to the *Anopheles gambiae* genome version AgamP4 (https://www.vectorbase.org/). Remaining reads were assembled into contigs and scaffolds using Velvet^75^ and SOPRA^76^. Filtered reads uniquely mapped to the *P. falciparum* reference genome were merged with assembled scaffolds to get all reference genomic regions matching sequences present in the Burkina dataset. This allowed us to confirm that 95.04% of the *de novo* assembly is mappable to the reference, and confirm the presence of nearly all annotated Pf3D7 genes, including *var* genes as well as other relevant clonally variant multigene families (*rifin, stevor*, and *pfmc-2TM*), (Figure S1). The percentage of the 3D7 genome reference covered by the *de novo* Burkina assembly was calculated using the intersectBed tool in Bedtools. Table S10 includes coverage data corresponding to CVGs. In order to estimate sequence similarity between Burkina isolates and the Pf3D7 reference genome sequence, variant calling was performed using SAM-tools in combination with vcflib (https://github.com/vcflib/vcflib) to obtain variants with a quality score greater than 20.

In order to examine coverage bias in the Burkina assembly, for example in telomeric and sub-telomeric regions that are AT-rich and repetitive, we first calculated theoretical mappability of the Pf3D7 reference genome. For this purpose, we used BEADS v2.1^77^. First, we obtained all possible 50 bp sequence tags from the *P. falciparum* 3D7 genome (PlasmoDB v25) and mapped them back against the same reference using Bowtie. Coverage plots were generated by counting the number of overlapping uniquely mapped tags in a 10 bp window and plotted them as a percent of maximum. The resulting mappability track was visualized together with the gene track for the 3D7 reference genome and the scaffolds of *de novo* assembly in IGV (Figure S2).

In addition, we performed further coverage analysis using deepTools^78^. First, we plotted the fraction of the genome covered by the ChIP-seq and input data (Figure S6A). Then, we applied the computeGCbias module of deepTools to quantify the G + C bias defined as ratio of the expected/observed GC fraction (GC fraction is defined as the number of G’s or C’s in a genome region of a given length) (Figure S6B). Finally, to test how the number of reads mapped might affect coverage of the ChIP-seq data we applied a 10% decrease scaling factor to ChIP samples and computed Pearson correlation coefficient between pairs of bam files (Figure S6C).

### ChIP-seq data analysis

After all quality controls described above, mapping of ChIP-sequencing reads was performed using Bowtie v1.1.1^79^ with default parameters, number of mismatches in the seed alignment set to 2, the trimming option set based on quality estimates above, and –m option set to 1 to report uniquely mapped alignments.

Peak calling was performed using MACS v1.4.2^80^,^81^. For this analysis, histone ChIPs and control inputs were down-scaled to the same number or reads. Parameters were set to a p value cutoff of 1 × 10^−5^, –keep-dup parameter of MACS set as default (2 duplicates retained), and the mfold parameter adjusted for each ChIP-seq data set. External ChIP-seq data^30^ of *P. falciparum* 3D7 parasite stages of the intra-erythrocytic life cycle were downloaded and subjected to the same analysis pipeline (Figure S3). For visualization purposes and to facilitate comparison between parasite stages, a common number of uniquely mapped reads were picked for each data set (0.5 M), and analyzed using MACS.

A recent study mapped transcription start sites in *P. falciparum* at high resolution and revealed that 81% of the TSSs were positioned less than 1000 bp upstream of the start codon, and more than half of these are located within a distance of 500 bp or less^39^,^41^. We thus used the gene ensemble available at PlasmoDB (Pf3D7 v25) for genome-wide analysis and considered the ATG ±1000 bp upstream as the putative promoter region. Heatmaps and profile plots for each histone modification were built using ngs.plot^82^ on duplicate-removed bam files. The resulting normalized (rpkm) and input corrected bin counts were used to estimate the average enrichment of each histone modification across various genomic features (genes, promoters and transcription binding site motifs). To verify that the patterns were robust, we built genome-wide histone maps but discarding genes located less than 1 kb from a neighboring gene. This analysis resulted in the same pattern of histone modification profiles (data not shown). Hierarchical clustering and heatmap generation on histone enrichment for different gene sets was implemented using the heatmap.2 function of the “ggplots” R package. Interval operations like intersect, merge, flank or slop were performed using BEDTools2.19.0^83^. We used SAMtools v1.4 (http://samtools.sourceforge.net) for SAM/BAM file conversion and manipulations, deduplication and to report read mapping statistics.

For the histone modification enrichment analysis of *var* genes, we used BEDTools2.19.0^83^ to obtain the number of reads overlapping promoter regions (±150 bp of the ATG). The resulting read counts were normalized, ChIP to noise signal corrected (ChIP/input ratio), and squared root transformed using R.

### RNA-seq data analysis

RNA-seq reads were trimmed at both ends based on the quality estimates for each sequence using the FASTX-Toolkit (http://hannonlab.cshl.edu/fastx_toolkit). RNA paired directional reads were mapped to the GTF annotation file of v25.0 of the *P. falciparum* genome using TopHat v2.0.13^84^. Reads were aligned using the option of library type set as first-strand for directional RNA-seq, segment-length after trimming, and the minimum and the maximum intron-length set to 5 and 1000, respectively, based on estimates obtained from a previous study^85^. The mean and standard deviations for the inner distance between mate pairs was empirically determined for each library using Picard Tools CollectInsertSizeMetrics (picard.sourceforge.net). Given the high polymorphic nature of the *P. falciparum* genome and the fact that this study deals with field parasite isolates, we tested the mapping specificity of sequencing reads to the reference assembly Pf3D7 by relaxing the number of mismatches. The modification of this parameter (up to 4 allowed mismatches) had no significant effect on the overall number of reads mapped. We used SAMtools v1.4 (http://samtools.sourceforge.net) for SAM and BAM file manipulation and conversion. Quantification and differential gene expression analysis were conducted using HTSeq/DESeq2 packages^86^. To count reads, HTSeq (http://www-huber.embl.de/users/anders/HTSeq/) configuration parameters were set for a strand-specific assay to separate between sense and antisense transcripts. The matrix of raw read counts was used as input for the R/Bioconductor DESeq2 package^86^, that performs library normalization and uses negative binomial generalized linear models to identify differentially expressed genes. Statistical significance was set to P adj <0.001 and >2-fold differential expression. In the case of *var* genes we employed the R/Bioconductor package DEXSeq^87^, which tests for differential expression at the exon level. The method uses generalized linear models to estimate an overall expression level for each exon, and then infers differential gene expression by assessing the changes of expression at the exon level between genes. Sets of differentially expressed genes between parasite stages were analyzed using Gene Ontology (GO) and Kyoto Encyclopedia of Genes and Genomes (KEGG) to identify significantly represented functional groups (P < 0.05). Gene annotation and functional enrichment analysis of GO terms were performed using the set of tools available at the PlasmoDB database.

Heatmaps of gene expression data were constructed using the “heatmap.2.” function implemented in the “gplots” package of R. Statistical tests and plots were performed in R 3.1.0 (http://www.r-228 project.org/) using Bioconductor (http://www.bioconductor.org).

For comparative purposes, gene expression data corresponding to RNA-seq normalized counts obtained from DESeq2 was log2 transformed and we added a pseudocount (0.1) to avoid dividing by 0. For ChIP-seq data, we performed square root transformation and used the value of enrichment level as a measurement of histone modification abundance. To explore the relationship between ChIP-seq signal and RNA levels in each stage of *P. falciparum* development in the mosquito, we first divided genes based on their expression into high, intermediate or low. Then we obtained the distribution of each histone modification enrichment levels in the  ±1 kb region surrounding the ATG translation start codon. To measure the quantitative association between histone modifications and mRNA levels we fitted a linear regression model for all genes using the R package lm, following similar studies by others^88^,^89^. For the linear regression model, we used the R2 decomposition implemented in the calc.relimp function of the *relaimpo* R package. The model considers gene expression as response and ChIP-seq enrichment levels (1 kb ± gene bodies) as covariates and calculated R squared, which gives a measure of the proportion of variance of gene expression that is explained by changes in histone modification abundance. We performed ANOVA analysis to test the linear model fit of each histone modification individually and the combined effect of multiple active histone marks (i.e. 2 or 3 histone mark models considering various combinations of H3K9ac, H3K27ac and H3K4me3). H3K9me3 was not considered in the combinatorial models.

### Motif analysis

In order to identify putative transcription factors responsible for changes in transcription during *P. falciparum* development, the set of significantly differentially expressed genes was subjected to motif search analysis using FIRE^37^. The FIRE algorithm compiles a list of candidate target genes associated with each predicted motif. These target genes share two characteristics: (i) at least one instance of the considered motif in their promoter regions and (ii) peak mRNA abundance levels within a particular stage of the *P. falciparum* transcriptome that is significantly enriched in genes whose promoters contain the motif. In order to investigate whether predicted binding sites for AP2 factors are among the motifs identified by FIRE, we performed similarity domain analysis with the TomTom tool (http://meme.nbcr.net/meme/doc/tomtom.html) using as reference the database of validated AP2 motifs previously published^33^. Sequence logos were constructed using WebLogo (http://weblogo.berkeley.edu). Cytoscape network analysis (http://www.cytoscape.org/) was used to investigate links between target genes identified by FIRE that have binding motifs for significantly enriched AP2 transcription factors and their expression levels in different stages during sporogonic development.

For the motif analysis at *var* genes we conducted motif scan using FIMO^90^ to validate the occurrence of the PF3D7_1466400 AP2 transcription factor binding sites on *var* gene sequences encompassing a 2 kb region upstream of the ATG start codon. In a subset of UPSB *var* genes, the motif is located near the ATG start codon^33^. We first examined histone modification profiles at the promoter by considering regions including 150 bp upstream and downstream of the ATG of all *var* genes. We then filtered *var* genes that have motifs for the PF3D7_1466400 AP2 transcription factor in this region. Among the set of genes that have the motif near the ATG is the active *var* gene PF3D7_1255200. We then used this subset of UPSB *var* genes for the differential enrichment analysis of histone modification profiles.

### Real-time quantitative PCR

Results obtained by RNA-seq were validated for a subset of *var* genes, including the active gene PF3D7_1255200. RNA was digested with DNase I (Invitrogen) according to the manufacturer’s instructions. Reverse transcription was performed using Superscript II RT (Invitrogen). Each 20 μl reaction mixture contained 0.5–1.0 μg of midgut or salivary gland purified RNA, 5× first strand buffer (250 μM Tris-HCl, 375 μM KCl, 15 μM MgCl_2_), 10 μM dNTP mix, 0.1 M DTT, 50 U Superscript II reverse transcriptase, 50 U RNase inhibitor, and 100 μM random hexamers. Amplification was performed using an Applied Biosystems 7500 Fast Real-Time PCR System. Reactions (12.5 μl) were performed in triplicate and consisted of 0.5 μl of cDNA, 0.2 μM of forward and reverse primers, and 5 μl of KAPA SYBR FAST qPCR Master Mix (KapaBiosystems). Cycling conditions were 10 min initial denaturation at 95 °C, followed by 40 cycles of 20 sec denaturation at 95 °C, 54–58 °C annealing and 30 sec extension at 60 °C. Melting curve analysis was performed to guarantee specificity of the template. The amount of each sequence in cDNA was determined relative to its level in a constant quantity of 3D7 strain gDNA and the amounts of cDNA and gDNA were normalized using the housekeeping gene S-adenosylmethionine synthetase (PF3D7_0922200). The 2−ΔΔCt CT method^91^ with improvements introduced by Pfaffl^92^ was used to account for PCR efficiency curves deviating from the theoretical 100% efficient reaction. The fold change between parasite stages was calculated for each gene by determining the difference in the Ct value for each time point. Transcription of the active *var* gene PF3D7_1255200 was confirmed in both oocysts and sporozoites. *var* product-specific amplification was confirmed by performing melting curves for each reaction, gel electrophoresis of expected sizes, and direct sequencing of amplification products.

## Human Subjects

This study involves human subjects. Participants were recruited from blood screening campaigns in local endemic areas of transmission near Bobo-Dioulasso, Burkina-Faso. Ethical approval was obtained from the Centre Muraz Institutional Ethics Committee (A003-2012/CE-CM). The protocol conforms to the declaration of Helsinki on ethical principles for medical research involving human subjects (version 2002) and informed written consent was obtained from all volunteers.

## Availability of Data and Material

ChIP-seq and RNA-seq data are deposited in the GEO database under accession number GSE68667.

## Additional Information

**How to cite this article**: Gómez-Díaz, E. *et al*. Epigenetic regulation of *Plasmodium falciparum* clonally variant gene expression during development in *Anopheles gambiae.* *Sci. Rep.* **7**, 40655; doi: 10.1038/srep40655 (2017).

**Publisher's note:** Springer Nature remains neutral with regard to jurisdictional claims in published maps and institutional affiliations.

## Supplementary Material

Supplementary Figures

Supplementary Table 1

Supplementary Table 2

Supplementary Table 3

Supplementary Table 4

Supplementary Table 5

Supplementary Table 6

Supplementary Table 7

Supplementary Table 8

Supplementary Table 9

Supplementary Table 10
