# Supplementary material for: Epigenetic regulation of Plasmodium falciparum clonally variant gene expression during development in Anopheles gambiae
Source: Sci Rep. 2017 Jan 16;7:40655. doi: 10.1038/srep40655 (PMC5238449; doi:10.1038/srep40655)
Supplement: Supplementary Figures [file srep40655-s1.pdf]

Epigenetic regulation of *Plasmodium falciparum* clonally variant gene expression during development in *An. gambiae*

Elena Gómez-Díaz, Rakiswendé S. Yerbanga, Thierry Lefèvre, Anna Cohuet, M. Jordan Rowley, Jean Bosco Ouedraogo, and Victor G. Corces

SUPPLEMENTAL TABLE LEGENDS

Table S1. Information on the donors and their gametocytemia (parasite density at the gametocyte/ring stage of *P. falciparum* field isolates), prevalence of infection (proportion of mosquitoes successfully infected at the oocyst stage), intensity of infection (density of occyst at day 7), and number of female mosquitoes dissected for each stage and experimental condition.

Table S2. Statistics of ChIP-seq and RNA-seq data processing and quality checks for gametocyte/ring, oocyst and sporozoite stages libraries.

Table S3. Differentially expressed genes in the in the oocyst and sporozoite stages and between the blood (gametocytes/rings) and the oocyst stages.

Table S4. GO analysis of oocyst specific and sporozoite specific genes listed in Table S3. Colors indicate oocyst (orange) and sporozoite (blue). Oocyst over-expressed genes are significantly enriched in functional categories associated with growth (ribosome structure and maturation), metabolism (starch and sucrose metabolism, purine and pyrimidine metabolism), and transcription and splicing. Sporozoite over-expressed genes are significantly enriched in proteins involved in host-parasite interactions and pathogenesis, signaling, cytoskeleton organization and biogenesis.

Table S5. Proportion of variance in gene expression levels explained (R squared) by each individual histone modification and the combinatorial models in each stage of *P. falciparum* development in the mosquito. ANOVA statistics of model fit are also shown for oocyst and sporozoite stages.

Table S6. Genes in the oocyst and the sporozoite stages that display significant H3K9me3 peaks (by MACS analysis) and high to medium levels of gene expression. Functional annotations (GOs for function, biological process and component terms), product description and enzyme EC number for each gene are listed. Colors indicate oocyst (orange) and sporozoite (blue).

Table S7. List of genes belonging to previously described CVG families (Rovira-Graells et al. 2012) showing significant changes in expression between mosquito parasite stages, and the corresponding differential enrichment in histone modifications (1 kb upstream ATG plus gene body). Genes encoding rRNAs and small non-coding RNAs were excluded from this analysis. Colors indicate oocyst (orange) and sporozoite (blue).

Table S8. Target genes for the predicted motifs identified in the set of differentially expressed genes in *P. falciparum* oocyst and sporozoite mosquito stages by FIRE analysis.

Table S9. Location of the [AG]C[AG]TGC[AGT] motif (similar to the binding site for the AP2 transcription factor PF14_0633) identified by FIMO analysis to be enriched in the 2 Kb 5' upstream region of *var* genes. The sites corresponding to the active PF3D7_1255200 *var* gene.

Table S10. Percentage of the reference (Pf3D7 v28) covered by "de novo" assembly generated from Burkina *Plasmodium* field isolates. List of genes include *var* , *rif*, *stevor* and *Pfmc-2TM* genes, as well as other clonally variant gene families.

SUPPLEMENTAL FIGURES

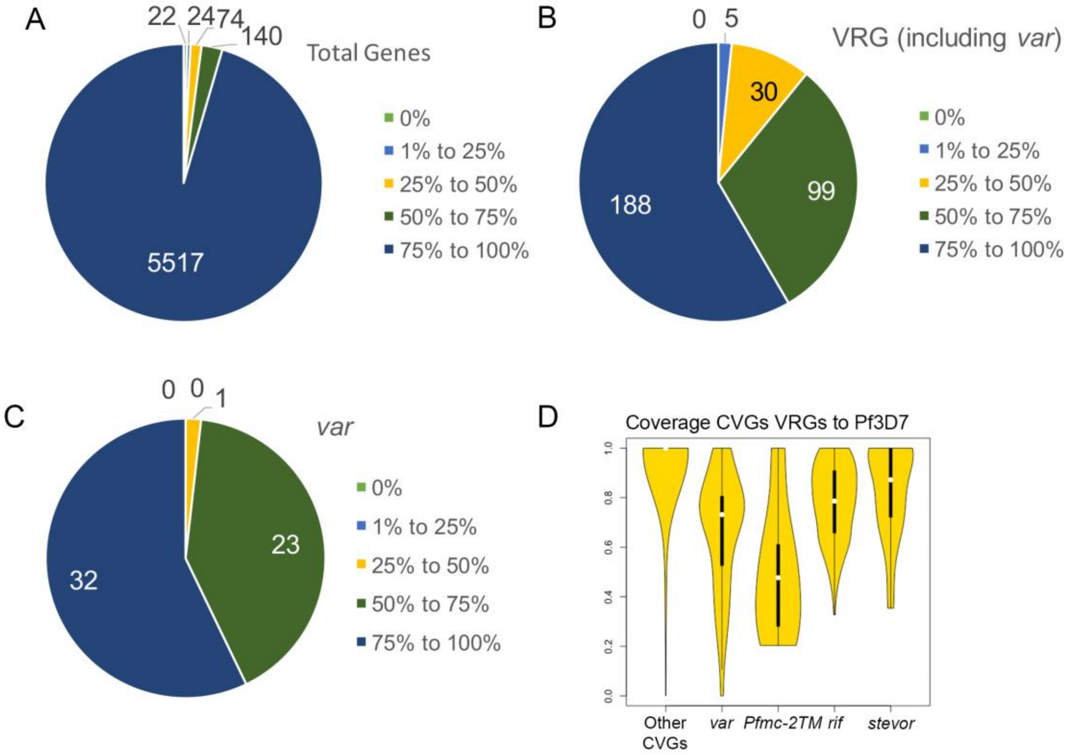

Figure S1. Conservation of the genome assembly generated *de novo* from Burkina field isolates with respect to the genome assembly of the reference clone 3D7.

A. Pie chart showing the percentage of genes (exons) in the 3D7 reference genome that are present in the Burkina assembly at various coverages. The majority of genes are covered at a percentage greater than 75%.

B. Same as above but centered on virulence genes, including *var*, *rif*, *stevor* and *Pfmc-2TM*. Coverage is reduced but it is still higher than 50% for the majority of virulence genes.

C. Same as above for the *var* genes only. All 3D7 reference variants are present at coverage higher than 50%.

D. Violin plot showing the distribution of coverage data for specific virulence genes and other clonally variant gene families analyzed in this study.

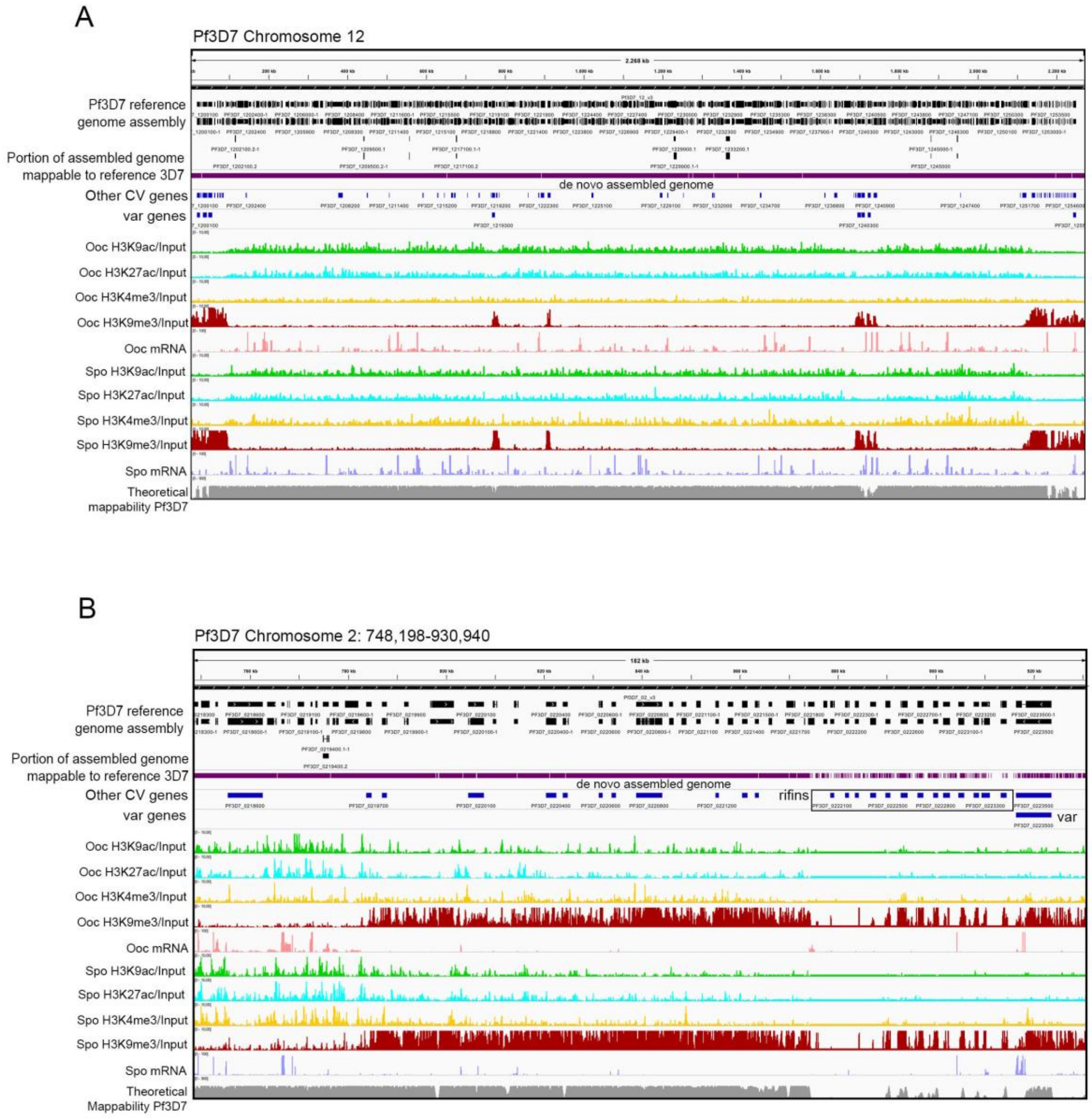

Figure S2. Coverage and mappability of the genome of *Plasmodium* field isolates from Burkina Faso assembled *de novo* compared to the 3D7 reference genome.
A. Example of a chromosome showing scaffolds of "*de novo*" assembly generated from the Burkina field isolates (purple track) that have coverage in the reference genome (black track). The location of *var* genes as well as other CVGs is indicated. Tracks for various histone modifications and mRNA levels for the oocyst and sporozoite parasite stages are also shown. The 100mer theoretical mappability track of the 3D7 reference genome indicates uniqueness of the region. Regions of low mappability, highly repetitive and AT-rich, are displayed as valleys where most reads are discarded during the mapping step in Bowtie. The assembled scaffolds in the Burkina samples with coverage in the 3D7 genome reference cover areas of high but also low mappability where most *var* genes are located.
B. Same as above but zooming in a smaller sub-telomeric region of chromosome 2 showing the position of a silenced *var* gene flanked by several *rif* genes in tandem.

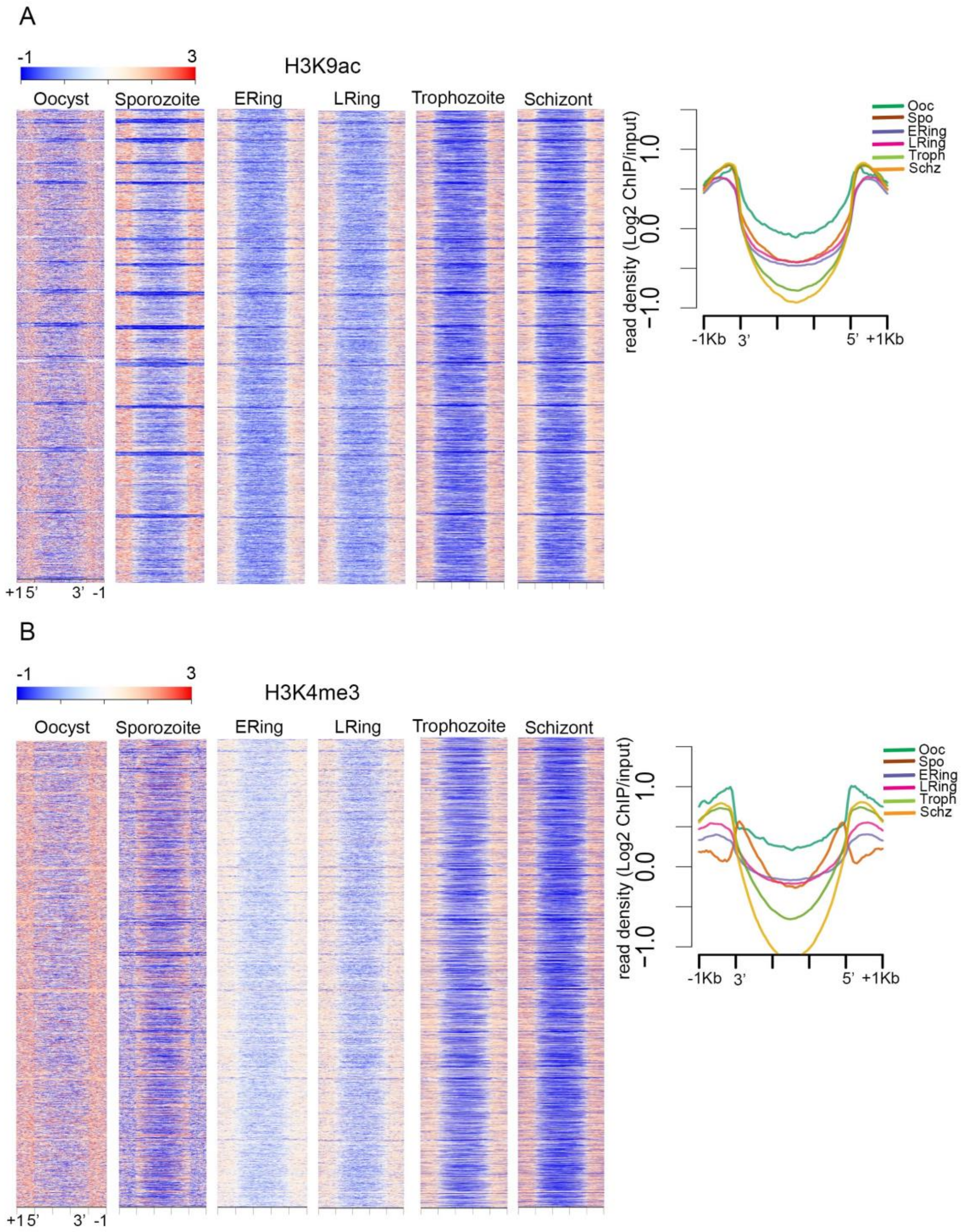

Figure S3. Histone modification profiles in *P. falciparum* genes at various stages of development in the mosquito and the human hosts (intra-erythrocytic cycle). Heatmaps correspond to ChIP-seq signal of H3K9ac (A) and H3K4me3 (B) for all coding genes annotated in the last version of the genome available at PlasmoDB (Pf3D7 v25). The region comprises 2000 bp upstream and downstream of the translation start and stop codons, respectively. The average profile plots show the density of normalized/input corrected reads for each histone modification and stage of development.

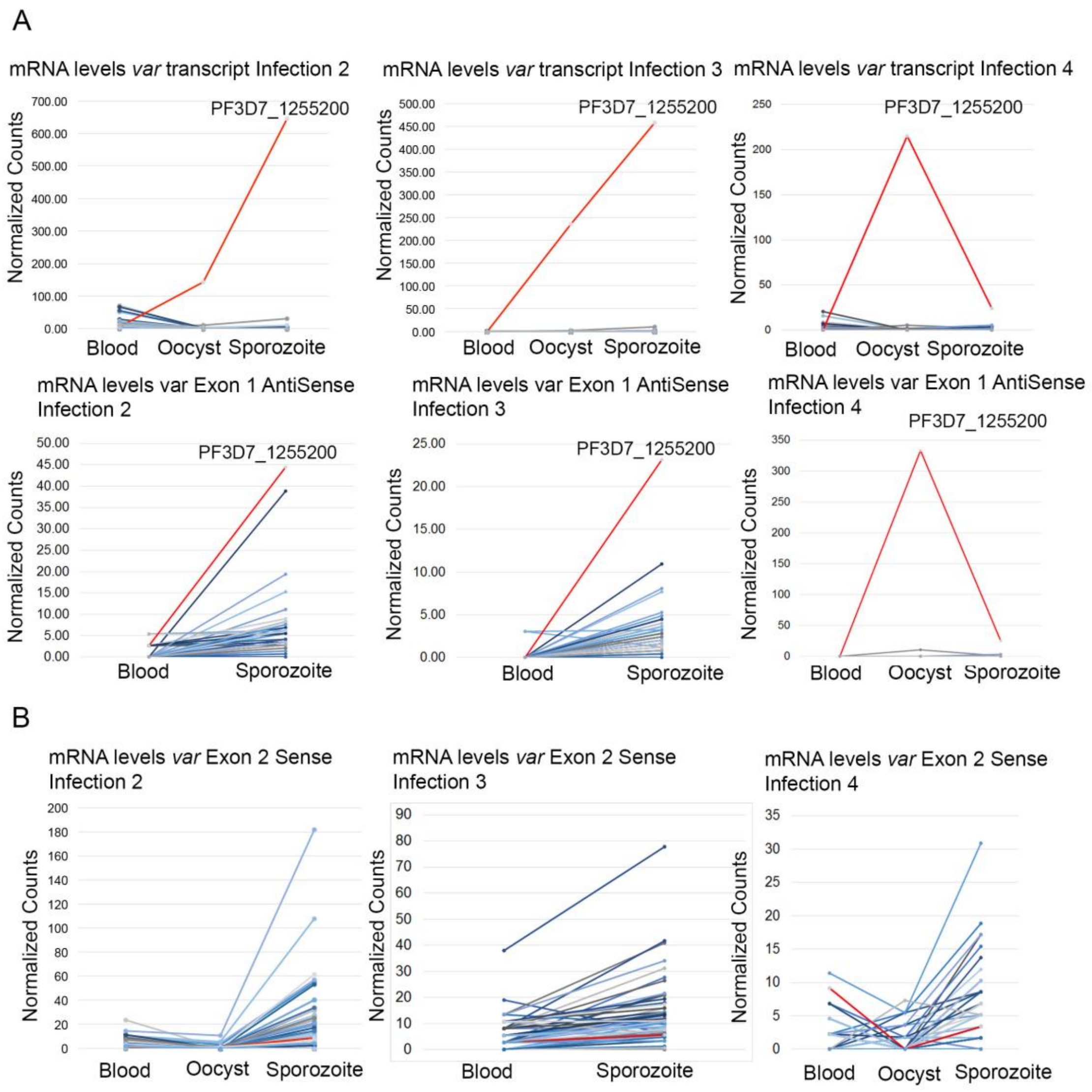

Figure S4. Gene expression patterns of *var* genes in the blood of human donors and during the sporogonic development of *P. falciparum* after infection.

A. Dynamics of exon 1 sense and antisense transcription for all *var* genes for infections 2, 3 and 4.

B. Dynamics of sense transcription of exon 2 for all *var* genes for infections 2, 3 and 4.

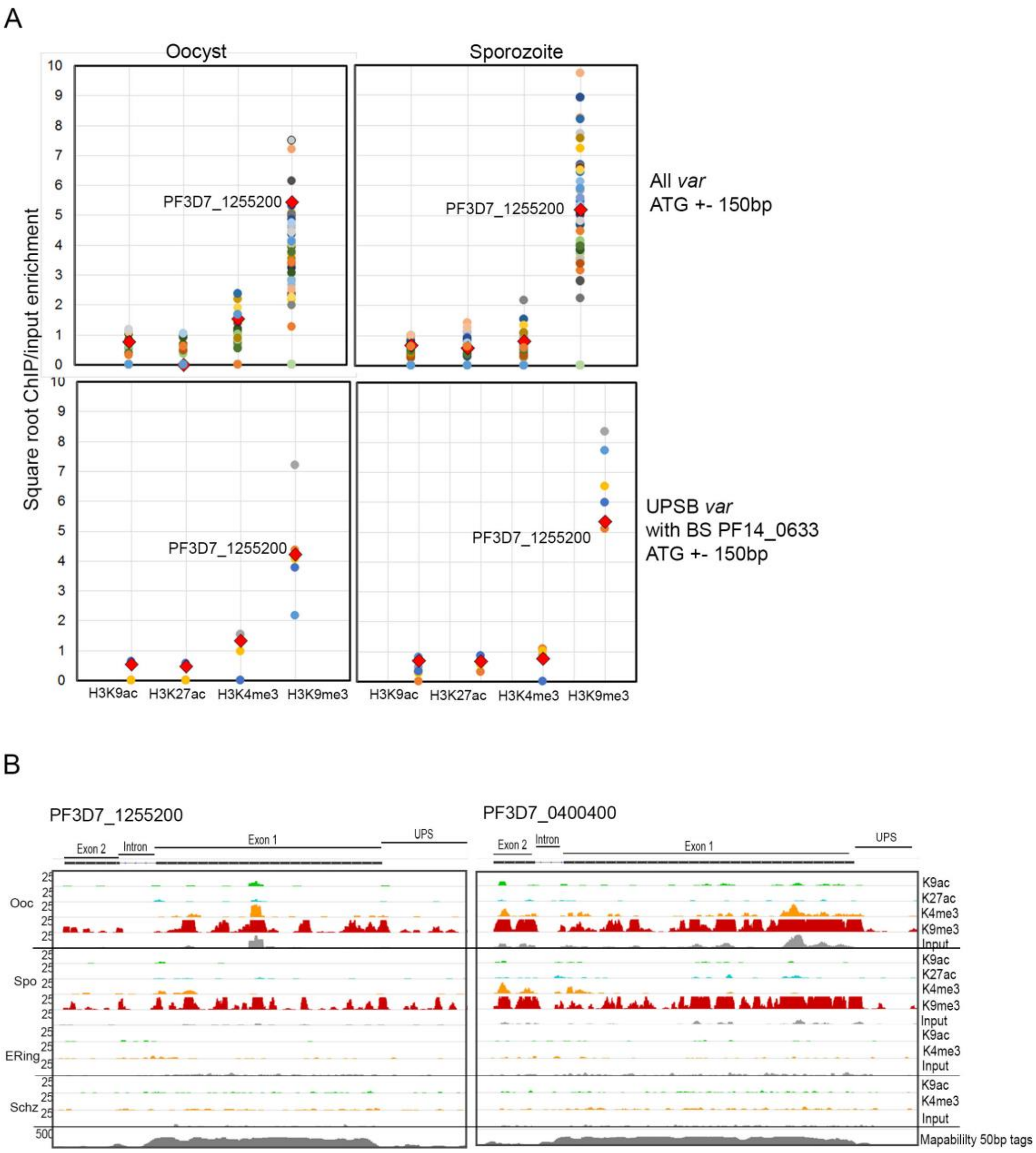

Figure S5. Histone modification profiles in the region of the ATG protein start codon (± 150 bp) of *var* genes during sporogonic development.

A. Top panels indicate the values of enrichment for each histone modification in the region of the ATG of all *var* genes in the oocyst and sporozoite stages. The lower panels indicate the same histone modification enrichment values for upsB type *var* genes, which contain the binding motif for the PF14_0633 AP2 transcription factor in this region. The active *PF3D7_1255200 var* gene is highlighted.

B. Examples of H3K9ac (green), H3K27ac (blue), H3K4me3 (yellow) and H3K9me3 (red) profiles for the active *PF3D7_1255200* and a silent *PF3D7_0400400 var* genes in oocysts and sporozoite stages. For comparison, profiles of H3K9ac and H3K4me3 obtained from various blood stages during the intra-erythrocytic cycle are included (data obtained from Bartfai et al. (2010)). The ChIP signal corresponds to the same number of reads between the parasite stages. The theoretical mapability track is shown for each *var* gene representing all possible 50 bp sequence tags from the *P. falciparum* genome effectively mapped.

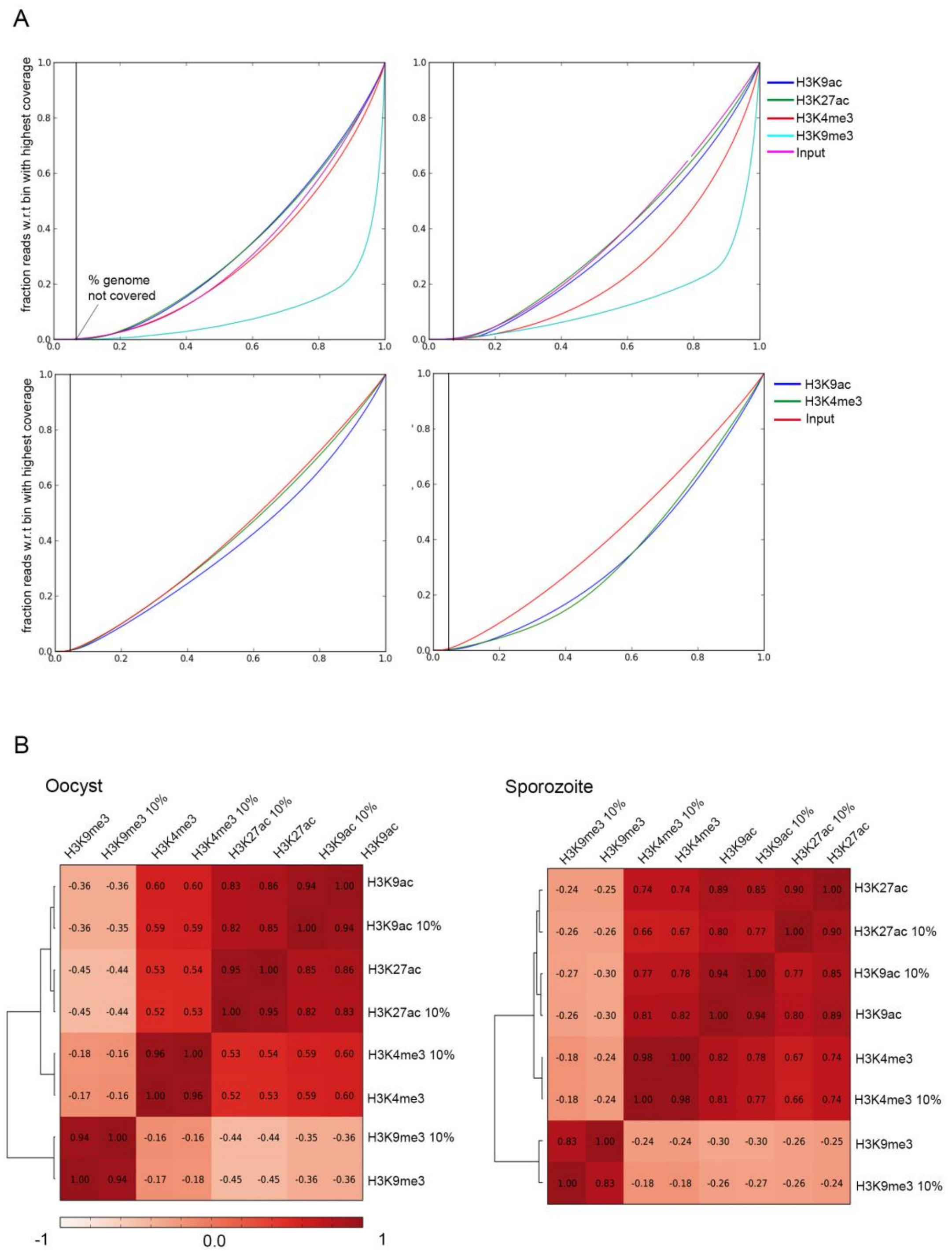

Figure S6. Additional quality control analysis of ChIP-seq data.
A. Cumulative sum of reads mapped per fraction of bins in the *P. falciparum* genome for each histone modification and the input controls at each stage of development. The value in the x-axis where the curves start to rise indicates the portion of genome covered by each ChIP-seq dataset. The shape of the curve reflects the distribution of the ChIP-seq signal along the genome (narrow and high vs. wide and not extremely high). For comparison, plots corresponding to the ring and trophozoite blood stages of data obtained from (Bartfai et al. 2010) are also shown.

B. Relationship between sequencing depth and coverage. The plot shows the correlation between ChIP-seq libraries for various histone modifications in each stage of development with the same libraries downscaled to a 10% number of total reads.
